# Anti-Amnesic Effect of Walnut via the Regulation of BBB Function and Neuro-Inflammation in Aβ_1-42_-Induced Mice

**DOI:** 10.3390/antiox9100976

**Published:** 2020-10-12

**Authors:** Jong Min Kim, Uk Lee, Jin Yong Kang, Seon Kyeong Park, Eun Jin Shin, Hyun-Jin Kim, Chul-Woo Kim, Mahn-Jo Kim, Ho Jin Heo

**Affiliations:** 1 Division of Applied Life Science (BK21 Plus), Institute of Agriculture and Life Science, Gyeongsang National University, Jinju 52828, Korea; myrock201@gnu.ac.kr (J.M.K.); kangjy2132@gmail.com (J.Y.K.); tjsrud2510@gmail.com (S.K.P.); jkeen7641@gnu.ac.kr (E.J.S.); hyunjkim@gnu.ac.kr (H.-J.K.); 2 Division of Special Purpose Tree, National Institute of Forest Science, Suwon 16631, Korea; rich26@korea.kr (U.L.); futuretree@korea.kr (C.-W.K.); otttr@korea.kr (M.-J.K.)

**Keywords:** walnut, tannin, amyloid beta, cognitive dysfunction, blood–brain barrier, neuroinflammation, Akt pathway

## Abstract

This study was conducted to assess the protective effect of walnut (*Juglans regia* L.) extract on amyloid beta (Aβ)_1-42_-induced institute of cancer research (ICR) mice. By conducting a Y-maze, passive avoidance, and Morris water maze tests with amyloidogenic mice, it was found that walnut extract ameliorated behavioral dysfunction and memory deficit. The walnut extract showed a protective effect on the antioxidant system and cholinergic system by regulating malondialdehyde (MDA) levels, superoxide dismutase (SOD) contents, reduced glutathione (GSH) contents, acetylcholine (ACh) levels, acetylcholinesterase (AChE) activity, and protein expression of AChE and choline acetyltransferase (ChAT). Furthermore, the walnut extract suppressed Aβ-induced abnormality of mitochondrial function by ameliorating reactive oxygen species (ROS), mitochondrial membrane potential (MMP), and ATP contents. Finally, the walnut extract regulated the expression of zonula occludens-1 (ZO-1) and occludin concerned with blood–brain barrier (BBB) function, expression of tumor necrosis factor-alpha (TNF-α), tumor necrosis factor receptor 1 (TNFR1), phosphorylated c-Jun N-terminal kinase (p-JNK), phosphorylated nuclear factor of kappa light polypeptide gene enhancer in B-cells inhibitor (p-IκB), cyclooxygenase-2 (COX-2), and interleukin 1 beta (IL-1β), related to neuroinflammation and the expression of phosphorylated protein kinase B (p-Akt), caspase-3, hyperphosphorylation of tau (p-tau), and heme oxygenase-1 (HO-1), associated with the Aβ-related Akt pathway.

## 1. Introduction

Alzheimer’s disease (AD) is caused by cerebral neurodegeneration and is accompanied by memory loss and cognitive dysfunction in elders [1]. Although the mechanism of neurodegeneration of AD is unknown, excessive oxidative stress and inflammatory toxicity derived from amyloid beta (Aβ) are considered to be among the main causes of AD [2]. In the amyloid metabolism pathway, Aβ produced by beta-secretase (BACE) and γ- secretase aggregates within the cell [3]. Aβ peptides are commonly found in the brain in the form of Aβ_1-40_ and Aβ_1–42_, and in particular, Aβ_1-42_ are more toxic than other species. Aβ_1-42_ is deposited in senile plaques and capillaries, resulting in greater toxicity [4]. The agglutination of Aβ_1–42_ causes the activation of inflammatory responses, production of oxidative stress, hyperphosphorylation of tau (p-tau) protein, and neuronal apoptosis in the cerebral neurons and glial cells [3]. This Aβ is reduced by various scavenging systems, such as neprilysin and insulin degrading enzyme (IDE), but these systems have been reported to be significantly reduced in AD patients [5].

Aβ promotes an increase in tumor necrosis factor-alpha (TNF-α) as an inflammatory necrosis factor, as well as the activity of phosphorylated c-Jun N-terminal kinase (p-JNK) [6]. The p-JNK phosphorylates insulin receptor substrate 1 (IRS-1) and stimulates phosphoinositide 3-kinase (PI3K)/protein kinase B (Akt) pathways [7]. Through this process, the activity of phosphorylated Akt (p-Akt) related to neuronal survival is inhibited, and it induces cytochrome C release from the mitochondria to the cytosol, thereby continuously causing an inflammatory response [8]. Additionally, this inflammatory response by Aβ induces microglia to stimulate the production of free radicals and the secretion of pro-inflammatory cytokines, such as interleukin 1 beta (IL-1β) and TNF-α [9]. 

In addition, increased Aβ is transported into the blood–brain barrier (BBB) through the receptor for advanced glycation end products (RAGE) and causes structural defects in the BBB, resulting in an inflammatory reaction [10]. The expression of RAGE in physiological conditions is normally low, but Aβ increases RAGE expression through the interaction between Aβ and RAGE. This reaction weakens BBB function by interfering with the tight junction [11]. RAGE–Aβ interaction promotes synaptic dysfunction and induces inflammation in glial cells [12]. This reaction increases the inflammatory response, persistent oxidative stress production, and apoptosis in neuronal cells, and ultimately initiates cognitive dysfunction, memory loss, and abnormal behavior [13]. Natural antioxidants help to ameliorate pathological conditions and prevent various diseases by alleviating disease progression [14]. On the other hand, because some antioxidants exhibit prooxidant activity under certain conditions, it is more important to consume natural products containing various antioxidants or health-functional foods that are proven to be non-toxic, rather than ingesting a large number of single antioxidants [14].

Walnut (*Juglans regia* L.) is a crop grown all over the world, and it is also cultivated in countries in East Asia, such as Korea [15]. Various nuts contain high concentrations of monounsaturated fatty acids, but in particular, walnuts are rich in polyunsaturated fatty acids, such as alpha-linolenic acid (ALA) [16]. Walnuts have various physiologically active compounds with excellent antioxidant activity, and in particular, ellagitannin-based tannins are as major bioactive substances [17]. Walnuts have been reported to have anti-inflammatory effects, as well as improving blood circulation and having protective effects against heart disease [18]. Also, walnuts inhibit liver fat accumulation, and reduce oxidative stress and apoptosis through a high-fat diet [19]. However, few studies have reported that the administration of walnuts improves cognitive dysfunction and inflammatory response by improving BBB function. Therefore, this study was conducted to evaluate the protective effect of walnuts against Aβ-induced cognitive dysfunction and memory deficits via regulation of the BBB function, neuroinflammation, and Aβ-related Akt pathways in institute of cancer research (ICR) mice.

## 2. Materials and Methods 

### 2.1. Chemicals

Dimethyl sulfoxide (DMSO), 5,5,6,6-tetrachloro-1,1,3,3-tetraethylbenzimidazolylcarbocyanine iodide (JC-1), sodium hydroxide, hydroxylamine, FeCl_3_, phenylmethanesulfonyl fluoride (PMSF), 2′,7′-dichlorofluorescein diacetate (DCF-DA), ο-phthalaldehyde, egtazic acid (EGTA), malate, pyruvate, phosphoric acid, metaphosphoric acid, thiobarbituric acid, Aβ_1-42_, and solvents were obtained from Sigma-Aldrich Chemical Co. (St. Louis, MO, United States). An ENLITEN adenosine triphosphate (ATP) assay system was purchased from Promega Corp. (Madison, WI, United States). ProtinEX animal cell/tissue, a tissue lysis buffer, was purchased from GeneAll Biotechnology (Seoul, Korea). A superoxide dismutase (SOD) assay kit was purchased from Dojindo Molecular Technologies, Inc. (Rockville, MD, United States).

### 2.2. Sample Preparation

The walnut (*Juglans regia* L.) used in this experiment was obtained from the Division of Special Forest Products, National Institute of Forest Science (Suwon, Korea). The walnut grown in Geumgok (Gimcheon, Korea) was selected through preliminary study compared to various cultivars (Figures S1 and S2). The sample was lyophilized using a vacuum tray drier (Operon, Gimpo, Korea) and stored at −20 °C. The sample was extracted with 50-fold, 60% ethanol at 40 °C for 2 h. The extracted sample was concentrated using a vacuum rotary evaporator (N-N series, Eyela Co., Tokyo, Japan), and lyophilized. The lyophilized extract of walnut was kept at −20 °C until use.

### 2.3. UPLC Q-TOF/MS^2^

To identify the bioactive compounds, extract of walnut was separated using n-hexane and water (50:50 *v*/*v*) to remove the fat. The mixture was centrifuged at 13,000× *g* for 10 min at 4 °C. The water solution was separated and lyophilized. The main physiologic compounds in extract of walnut were analyzed using an ultra-performance, liquid chromatography–ion mobility separation–quadrupole time of flight/tandem mass spectrometry (UPLC IMS Q-TOF/MS^2^; Vion, Waters Corp., Milford, MA, United States). UPLC separation was investigated with an ACQUITY UPLC BEH C_18_ column (2.1 × 100 mm, 1.7 μm particle size; Waters Corp.). The flow rate was performed 0.35 mL/min. The mobile phases were composed as solvent A (0.1% formic acid in distilled water) and solvent B (0.1% formic acid in acetonitrile), and analysis conditions were as follows: a gradient elution of 1% B at 0–1 min, 1–100% B at 1–7 min, 100% B at 7–8 min, 100–1% B at 8–8.2 min, and 1% B at 8.2–10 min. The conditions of negative electrospray ionization (ESI) were as follows: ramp collision energy, 10–30 V; capillary voltage, 2.5 kV; source temperature, 100 °C; desolvation temperature, 400 °C; cone voltage, 40 V; mass range, 50–1500 m/z. The data from the UPLC were analyzed using MarkerLynx software (Waters Corp.).

### 2.4. Animal Design

**Figure 1:**
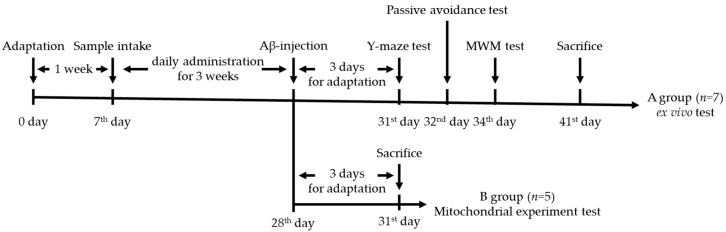
Experimental design of the in vivo test for amyloid beta (Aβ)-induced mice.

The male ICR mice (4 weeks old) were purchased from Samtako (Osan, Korea). The experimental animals were divided to four groups: NC group (vehicle-intracerebroventricularly (i.c.v.) injected/vehicle-administration), Ab group (Aβ_1-42_-injected/vehicle oral administration), and walnut extract (WE) 10 and WE 20 groups (Aβ_1-42_-injected/WE 10 and 20 mg/kg of body weight oral administration, respectively). The sample was intragastrically administrated into the stomach using the stomach tube daily for 3 weeks. After the sample administration, Aβ_1-42_ was injected as 410 pM in 10 μL saline using a 25 μL Hamilton microsyringe combined with a 26 gauge needle, according to the ethical guidelines. Aβ_1-42_ dissolved in 0.9% sterile saline solution with 1% ammonia solution was incubated for 4 days at 37 °C. Also, after injection, animals were individually housed in cages for 3 days for healing. All animal experiments received the approval of the Animal Care and Use Committee of Gyeongsang National University (certificate: GNU-181019-M0054), and were performed according to the provisions of Policy of the Ethical Committee of the Ministry of Health and Welfare, Republic of Korea. The experiment design is presented in Figure 1.

### 2.5. Behavioral Tests

Three days after the Aβ_1-42_ injection, behavioral tests were investigated to evaluate the anti-amnesic effect of walnut using a Y-maze test, passive avoidance test, and Morris water maze test.

#### 2.5.1. Y-maze Test

To evaluate the spontaneous alternation behavior, a Y-maze test was conducted [20]. This maze was made of black acrylic plate (length = 33 cm; height = 15 cm; width = 10 cm). The experimental animals were located at the end of the designated arm, and these mice were allowed to freely move in the maze for 8 min. The movement and path of the experimental animals were recorded using a video motion recognition system (Smart 3.0, Panlab, Barcelona, Spain).

#### 2.5.2. Passive Avoidance Test

To assess short-term memory, a passive avoidance test was performed using a shuttle box [21]. The test device consisted of a grid floor divided into bright and dark squares. First, the experimental animals were located in an illuminated square, and after 60 s, the door between the squares was opened. When the four feet of the experimental animals had entered the other square, a foot electric shock could be applied (0.5 mA, 3 s), and the time of first latency was recorded. After 24 h, the step-through latency time to re-enter the dark square was recorded (maximum time: 300 s).

#### 2.5.3. Morris Water Maze Test

To estimate long-term memory and spatial learning ability, a Morris water maze (MWM) test was performed [22]. The MWM circular pool (diameter 90 cm, height 30 cm) was divided into quadrants: the N, S, E, and W zones. The water in the MWM was diluted with squid ink, and a platform 1 cm below the surface of the water was located on one side of the quadrant. The experimental animals were allowed to swim, and they were trained repeatedly four times a day, each time with different positions. The movements of experimental animals were recorded using a SMART video tracking system (Smart 3.0, Panlab). Four training trials were investigated for each animal to swim and escape by repeating four times a day. Lastly, a probe trial was conducted without the platform for 90 s, and the time they stayed in the W zone was recorded.

### 2.6. Preparation of Tissue

After the behavior tests, animals were fasted for 12 h, and then sacrificed using CO_2_ inhalation for ex vivo tests. The collected brain tissues were homogenized using a bullet blender (BBY24M, Next Advance Inc., Averill Park, NY, United States) with phosphate-buffered saline (PBS) for the SOD, malondialdehyde (MDA), acetylcholine (ACh), and acetylcholinesterase (AChE) assays, and with 10 mM phosphate buffer with 1 mM EDTA (pH 6.7) for a reduced glutathione (GSH) assay at 4 °C. The cerebral protein concentration was measured with a Bradford protein assay [23].

### 2.7. Antioxidant System

#### 2.7.1. SOD Levels

To measure cerebral SOD levels, the homogenized tissue was spun down at 400× *g* at 4 °C for 10 min. The pellets in five-fold of ice-cold, 1× cell extraction buffer (10% SOD buffer, 0.4% (*v*/*v*) Triton X-100, and 200 μM phenylmethane sulfonylfluoride) were mixed at 10,000× *g* at 4 °C for 10 min. The measurement of cerebral SOD levels was conducted using commercial SOD kit (Sigma-Aldrich Chemical Co.), according to the provided protocol.

#### 2.7.2. Reduced GSH Levels

To measure reduced GSH levels, homogenized tissue was centrifuged at 10,000× *g* at 4 °C for 15 min. Then 5% metaphosphoric acid was reacted with the supernatant to remove the interference protein by spinning down at 2000× *g*. After that, 0.26 M tris-HCl (pH 7.8), 0.65 N NaOH, and 1 mg/mL ο-phthalaldehyde were mixed with supernatant at room temperature for 15 min. Fluorescence was measured at wavelengths of 320 nm (excitation filter) and 420 nm (emission filter) using a fluorometer (Infinite F200, Tecan Co., San Jose, CA, United States) [24].

#### 2.7.3. MDA Levels

To evaluate the cerebral MDA levels, homogenized tissue was mixed with 1% phosphoric acid and 0.67% thiobarbituric acid 95 °C for 1 h. The mixture was centrifuged at 2500× *g* for 10 min, and the absorbance was measured at 532 nm [25].

### 2.8. Cholinergic System

#### 2.8.1. ACh Levels

To measure the ACh levels, the homogenized tissue was centrifuged at 14,000× *g*. This supernatant was reacted with alkaline hydroxylamine reagent (3.5 N sodium hydroxide and 2 M hydroxylamine in HCl) at room temperature for 1 min, and 0.5 N HCl (pH 1.2) and 0.37 M FeCl_3_ in 0.1 N HCl were reacted with mixture. The absorbance was immediately measured at 540 nm [26].

#### 2.8.2. AChE Activities

To assess the AChE activity, the same supernatant as above was used for enzyme experiments. The supernatant was reacted with 50 mM sodium phosphate buffer (pH 8.0) at 37 °C for 15 min. After adding an Ellman’s reaction mixture, the absorbance was measured at 405 nm [27].

### 2.9. Mitochondrial Function

#### 2.9.1. Extration of Mitochondria 

The mitochondrial extraction method was performed according to Brown et al. Whole cerebral tissue was homogenized in a mitochondria isolation (MI) buffer (215 mM mannitol, 75 mM sucrose, 0.1% BSA and 20 mM HEPES sodium salt (pH 7.2)) with 1 mM EGTA. This homogenate was spun down at 1300× *g* for 5 min at 4 °C. The supernatant was re-centrifuged at 13,000× *g* for 10 min at 4 °C, and supernatant was discarded to remove the synaptosome. Continuously, the remaining pellet was mixed with MI buffer containing 0.1% digitonin and centrifuged at 10,000× *g* for 10 min at 4 °C. The pellet was added to the MI buffer to measure the mitochondrial function [28].

#### 2.9.2. Mitochondrial ROS Contents

To investigate the mitochondrial reactive oxygen species (ROS) levels, the isolated mitochondrial extract was incubated with a KCl-based respiration buffer (125 mM potassium chloride, 2 mM potassium phosphate monobasic, 20 mM HEPES, 1 mM magnesium chloride, 500 μM EGTA, 2.5 mM malate, and 5 mM pyruvate) and DCF-DA for 20 min. After incubation, fluorescence was measured at a wavelength of 485 nm (excitation filter) and 530 nm (emission filter) using a fluorometer (Infinite F200) [28].

#### 2.9.3. Mitochondrial Membrane Potential

To assess the mitochondrial membrane potential (MMP), the isolated mitochondria extract was mixed with 1 mM JC-1 in MI buffer containing 5 mM pyrivate and 5 mM malate. This mixture was reacted at room temperature for 20 min, and fluorescence was measured at a wavelength of 530 nm (excitation filter) and 590 nm (emission filter) using a fluorometer (Infinite F200) [28].

#### 2.9.4. Mitochondrial ATP Contents

ATP contents were evaluated using a commercial ATP bioluminescence assay kit (Promega Corp.) according to the manufacturer’s protocol. The ATP content was calculated according to a standard curve.

### 2.10. Western Blot 

The cerebral tissues were homogenized in ice-cold extraction solution (GeneAll Biotechnology, Seoul, Korea) with 1% protease inhibitor cocktail. The supernatants centrifuged at 13,000× *g* for 10 min at 4 °C were used to investigate the protein expression level. The proteins were separated by SDS-PAGE gel and transferred to the polyvinylidene difluoride (PVDF) membrane. The membranes were reacted in primary antibodies at 4 °C for 12 h and reacted with secondary antibodies at room temperature for 1 h. For chemi-luminescence detection, the immune complexes were detected using a Western blot imager (iBright Imager, Thermo-Fisher Scientific, Waltham, MA, United States). The density level of protein expression was calculated using image analyzer (ImageJ software, National Institutes of Health, Bethesda, MD, United States). Antibody information is presented in Table 1.

### 2.11. Statistical Analysis

All results were presented as mean ± standard deviation (SD). The statistical analysis was determined by one-way analysis (ANOVA) and determined using the Duncan’s new multiple-range test (*p* < 0.05) of SAS ver. 9.4 (SAS Institute Inc., Cary, NC, United States). Data were statistically represented as significantly different from the NC group (*) and significantly different from the Ab group (^#^), respectively (* and ^#^ *p* < 0.05, ** and ^##^ *p* < 0.01).

## 3. Results

### 3.1. UPLC Q-TOF/MS^2^

**Figure 2:**
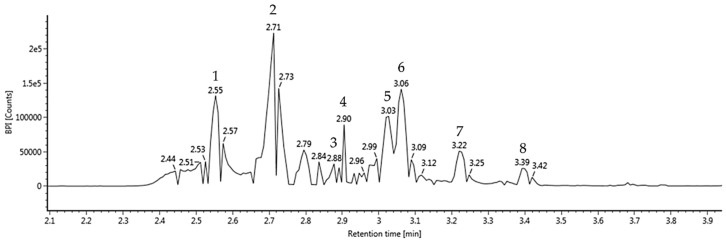
Ultra-performance, liquid chromatography–ion mobility separation–quadrupole time of flight/tandem mass spectrometry (UPLC Q-TOF/MS^2^) chromatographic profile of walnut extract.

The bioactive compounds of the walnut extract were qualitatively identified using UPLC IMS Q-TOF/MS^2^ analysis (Figure 2 and Table 2). The ESI-MS^2^ spectra were continuously obtained in negative ion mode (M−H)^−^ as compound 1: 783 *m*/*z* (retention time (RT): 2.55 min); compound 2: 783 *m*/*z* (RT: 2.71 min); compound 3: 951 *m*/*z* (RT: 2.88 min); compound 4: 785 *m*/*z* (RT: 2.90 min); compound 5: 935 *m*/*z* (RT: 3.03 min): compound 6: 433 *m*/*z* (RT: 3.06 min); compound 7: 1:085 *m*/*z* (RT: 3.22 min); and compound 8: 592 *m*/*z* (RT: 3.39 min). When the main fragments were compared with a previous study, these peaks were identified as a pedunculagin/casuariin isomer (bis-HHDP–glucose) (compound 1), pedunculagin/casuariin isomer (bis-HHDP–glucose) (compound 2), praecoxin A/platycariin isomer (trigalloyl-HHDP–glucose) (compound 3), tellimagrandin I isomer (digalloyl-HHDP–glucose) (compound 4), casuarinin/casuarictin isomer (compound 5), ellagic acid pentoside (compound 6), eucalbanin A/cornusiin B isomer (compound 7), and glansreginin A (compound 8), respectively [29].

### 3.2. Behavioral Tests

**Figure 3:**
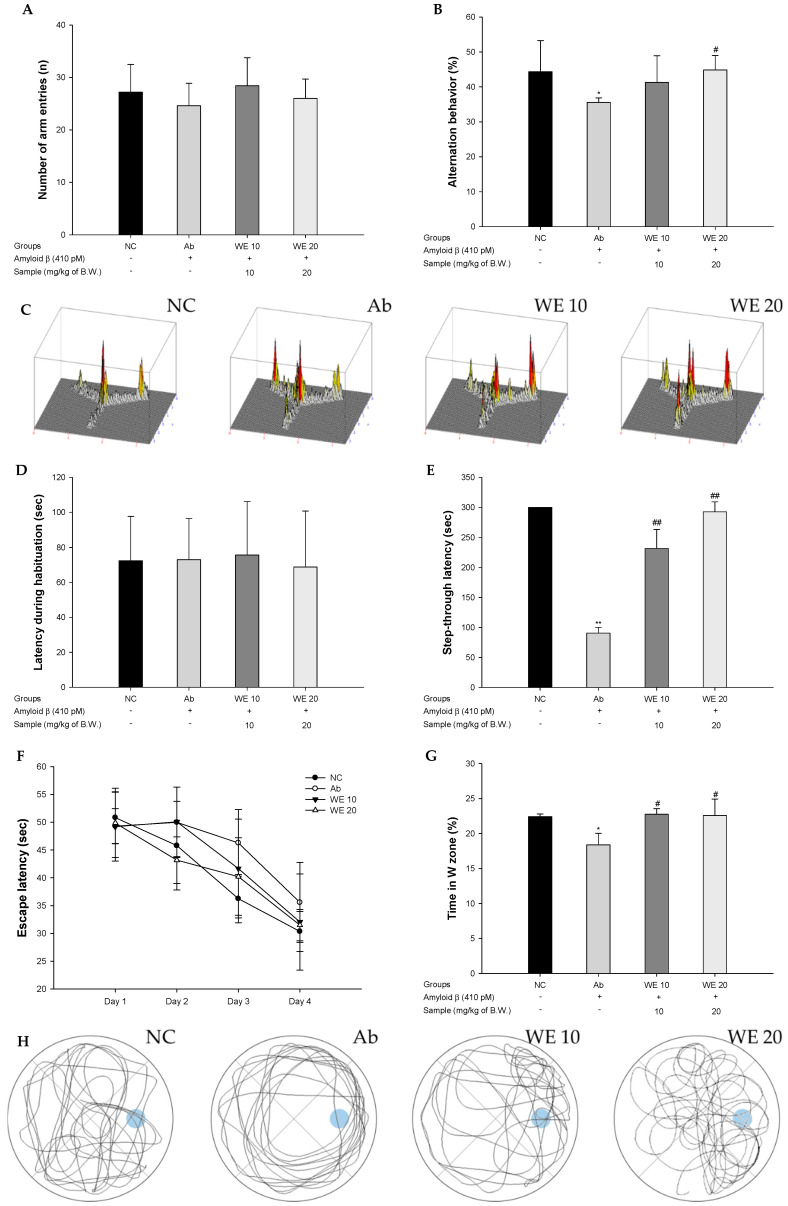
Protective effect of walnut (*Juglans regia* L.) extract in Aβ-induced mice: (**A**) Spontaneous alternation behavior; (**B**) number of arm entries; (**C**) three-dimensional (3D) moving routes; (**D**) latency during habituation; (**E**) step-through latency; (**F**) escape latency in the training trial; (**G**) retention time on W zone in the probe trial; and (**H**) path tracing of each groups in the probe trial. Results shown are means ± SD (*n* = 7). Data are statistically represented at * = significantly different from the NC group, and ^#^ = significantly different from Ab group; * and ^#^ *p* < 0.05, ** and ^##^ *p* < 0.01.

To confirm spatial learning and memory function, the Y-maze test was conducted (Figure 3A–C). The Ab group showed a significant decrease in spontaneous alternation behavior (35.58%) compared to the NC group (44.36%) (Figure 3B). However, the behavior of the WE groups (41.27% and 44.85%; WE 10 and WE 20, respectively) improved. In particular, the WE 20 group was significantly restored compare to the Ab group. The number of arm entries of all the groups was not statistically different (Figure 3A). In the results of path tracing, the Ab group showed irregular movements, but the WE groups were similar to the NC group (Figure 3C). 

To estimate short-term memory ability, the passive avoidance test was performed (Figure 3D,E). The first step-through latency showed no significant differences between all the groups (Figure 3D). In the trial test, the step-through latency of the Ab group (90.60 s) was reduced compared with the NC group (300.00 s) (Figure 3E). However, that of the WE groups was considerably ameliorated compared to the Ab group (231.75 s and 292.60 s; WE 10 and WE 20, respectively).

To measure spatial learning acquisition and long-term memory, the MWM test was conducted (Figure 3F–H). In the hidden trial, the escape latency of the Ab group (35.58 s) was reduced compared with that of the NC group (30.36 s) (Figure 3F). On the other hand, WE groups presented decreased escape latency through the administration (32.05 s and 31.49 s; WE 10 and WE 20, respectively). In the probe test, the retention time in the W zone for the Ab group (18.37%) decreased compared with the NC group (22.41%) (Figure 3G). However, the WE groups showed significantly increased retention times (22.75% and 22.58%; WE 10 and WE 20, respectively) compared to the Ab group. In the results of path tracing, the movements of the Ab group showed reduced retention time in the W zone compared with the NC group (Figure 3H). However, the WE groups showed improved retention time in the W zone.

### 3.3. Antioxidant System

**Figure 4:**
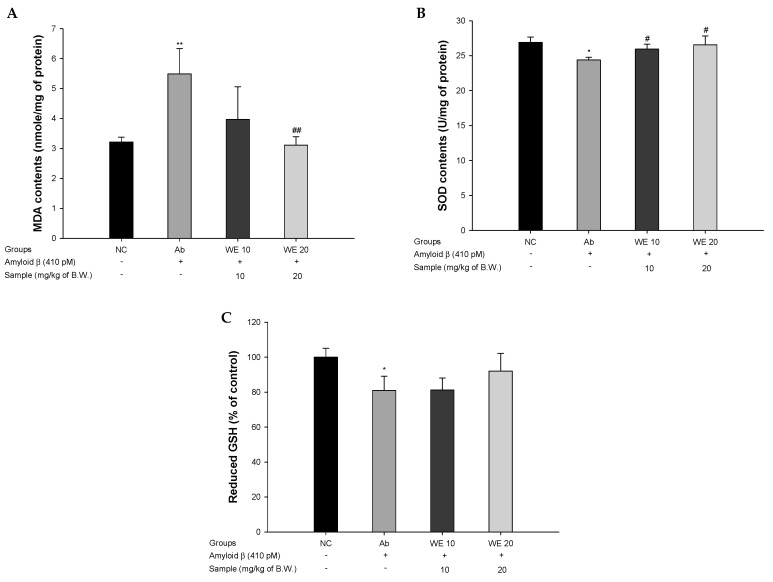
Protective effect of walnut (*Juglans regia* L.) extract on Aβ-induced biochemical changes related with antioxidant systems: (**A**) malondialdehyde (MDA) levels, (**B**) superoxide dismutase (SOD) levels, and (**C**) reduced glutathione (GSH) levels in mice brain tissues. Results shown are means ± SD (*n* = 5). Data are statistically represented at * = significantly different from the NC group; # = significantly different from Ab group; * and ^#^ *p* < 0.05, ** and ^##^ *p* < 0.01.

To evaluate the antioxidant system in Aβ_1-42_-induced mice, MDA levels, SOD levels, and reduced glutathione GSH levels were assessed (Figure 4). The MDA of the Ab group (5.49 nmol/mg of protein) was significantly produced compared to the NC group (3.22 nmol/mg of protein). On the other hand, the administration of walnut extract showed a reduced MDA level in the WE groups (3.97 nmol/mg of protein and 3.11 nmol/mg of protein; WE 10 and WE 20, respectively). In particular, those of the WE 20 group was significantly reduced compare to Ab group. The SOD levels of the Ab group (24.39 U/mg of protein) decreased compared with the NC group (26.92 U/mg of protein). The SOD levels of both WE groups (25.94 U/mg of protein and 26.57 U/mg of protein; WE 10 and WE 20, respectively) were significantly increased compared with the Ab group. The GSH levels of the Ab group (80.92% of control) was reduced compared to that of the NC group (100% of control). However, the reduced GSH levels of the WE groups (81.16% of control and 91.99% of control; WE 10 and WE 20, respectively) were higher compared to the Ab group. In particular, those of the WE 20 group was considerably increased compare to Ab group.

### 3.4. Cholinergic System

**Figure 5:**
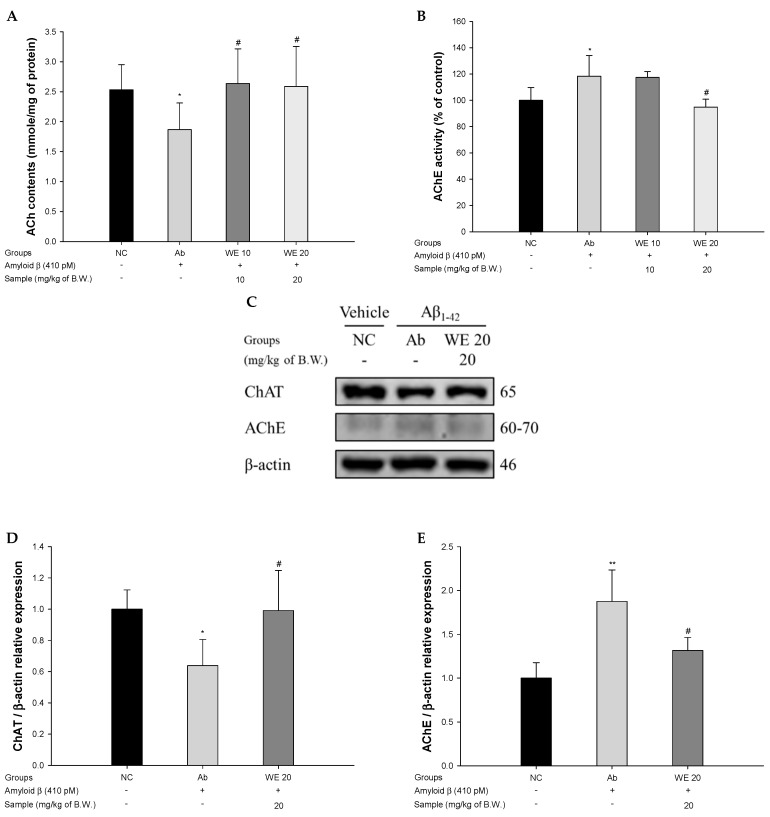
Protective effect of walnut (*Juglans regia* L.) extract on Aβ-induced cholinergic dysfunction. (**A**) acetylcholine (ACh) levels, (**B**) acetylcholinesterase (AChE) activities, (**C**) protein expression levels, (**D**) representative Western blots for the total protein and expression of choline acetyltransferase (ChAT) in mice brain tissues, and (**E**) representative Western blots for the total protein and expression of AChE in mice brain tissues. Results shown are means ± SD (A,B: *n* = 5; C–E: *n* = 3). Data are statistically represented at * = significantly different from the NC group; ^#^ = significantly different from Ab group; * and ^#^ *p* < 0.05, ** *p* < 0.01.

To assess the function of the cholinergic system in Aβ_1-42_-induced mice, ACh levels, AChE activity, and expression levels of choline acetyltransferase (ChAT) and AChE were conducted (Figure 5). The ACh levels of the Ab group (1.87 mmol/mg of protein) decreased compared to that of the NC group (2.53 mmol/mg of protein) (Figure 5A). However, the intake of walnut extract showed significantly increased ACh levels (2.64 mmol/mg of protein and 2.59 mmol/mg of protein) compared to the Ab group. The AChE of the Ab group (118.34%) increased compared to the NC group (100%) (Figure 5B). However, intake of WE ameliorated AChE activity (117.42% and 94.87% for WE 10 and WE 20, respectively) compared to the Ab group. Therefore, the intake of walnut extract had a significant effect in regulating ACh content and AChE activity. The expression of ChAT and AChE is shown in Figure 5C. The ChAT expression level in the Ab group was downregulated by 36.17% compared to the NC group (Figure 5D). However, the WE 20 group showed increased ChAT expression (55.15%) compared to the Ab group. The AChE expression level in the Ab group was upregulated by 87.44% compared to the NC group (Figure 5E). However, the WE 20 group showed inhibited AChE expression (29.87%) compared to the Ab group. The WE 20 group showed a considerable ameliorating effect of expression levels of ChAT and AChE compare to the Ab group.

### 3.5. Mitochondrial Function

**Figure 6:**
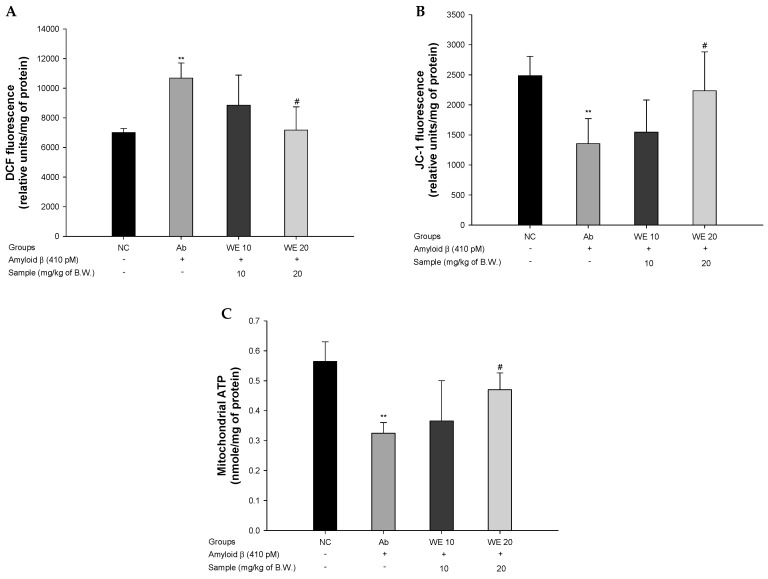
Protective effect of walnut (*Juglans regia* L.) extract on Aβ-induced mitochondrial dysfunction: (**A**) reactive oxygen species (ROS) levels, (**B**) mitochondrial membrane potential (MMP) levels, (**C**) ATP contents of the mitochondria in mice brain tissues. Results shown are means ± SD (*n* = 5). Data are statistically represented at * = significantly different from the NC group; ^#^ = significantly different from Ab group, respectively; ^#^ *p* < 0.05, ** *p* < 0.01.

To assess mitochondrial function in Aβ_1-42_-induced mice, ROS production, MMP, and ATP levels were evaluated (Figure 6). The ROS production of the Ab group (10684.25 relative units/mg of protein) increased compared to that of the NC group (7013.25 relative units/mg of protein) (Figure 6A). However, the WE groups (8854.32 relative units/mg of protein and 7175.47 relative units/mg of protein for the WE 10 and WE 20 groups, respectively) showed considerably decreased DCF production compared with the Aβ group. The MMP of the Ab group (1357.57 relative units/mg of protein) was reduced in comparison with the NC group (2486.25 relative units/mg of protein) (Figure 6B), whereas that of the WE groups (1545.25 relative units/mg of protein and 2235.12 relative units/mg of protein for the WE 10 and WE 20 groups, respectively) improved compared to the Ab group. The ATP content of the Ab group (0.32 nmole/mg of protein) decreased compared to that of NC group (0.56 nmole/mg of protein) (Figure 6C). However, that of the WE groups (0.37 nmole/mg of protein and 0.47 nmole/mg of protein for WE 10 and WE 20, respectively) was restored compared to the Ab group. There was no significant difference in the WE 10 group, but the WE 20 group showed a significantly protective effect on mitochondrial activity compare to Ab group.

### 3.6. Blood–Brain Barrier (BBB) Function

**Figure 7:**
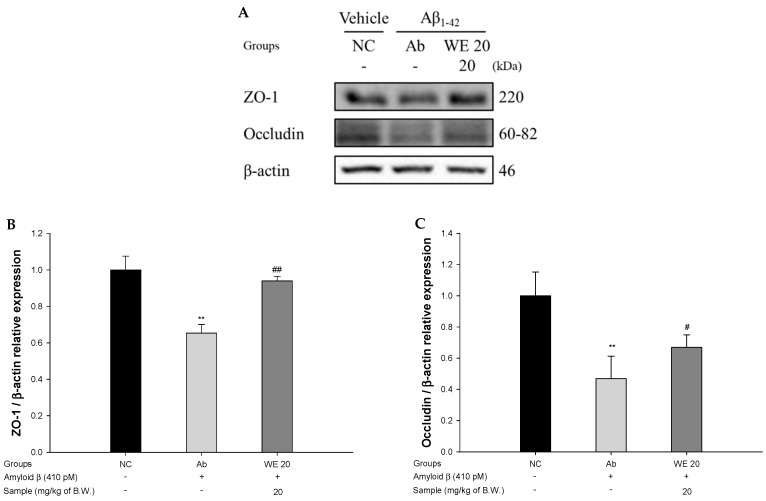
Protective effect of walnut (*Juglans regia* L.) extract on Aβ-induced blood brain barrier (BBB) dysfunction: (**A**) protein expression levels; (**B**) representative Western blots for total protein and expression of zonula occludens-1 (ZO-1) (**C**) and occludin in mice brain tissues. Result shown are means ± SD (*n* = 3). Data are statistically represented at * = significantly different from the NC group; ^#^ = significantly different from Ab group; ^#^ *p* < 0.05, ** and ^##^ *p* < 0.01.

To evaluate the protective effect of the walnut extract on Aβ_1-42_-induced mice via the regulation of BBB function, the protein expressions of zonula occludens-1 (ZO-1) and occludin were measured (Figure 7). The expression levels of ZO-1 and occludin in the Ab group were significantly reduced (34.70% and 53.09%, respectively) compared to the NC group. However, the administration of the walnut extract significantly inhibited the decreased expression of ZO-1 and occludin (43.91% and 42.68%, respectively) compared to the Ab group.

### 3.7. Neuroinflammation Pathway

**Figure 8:**
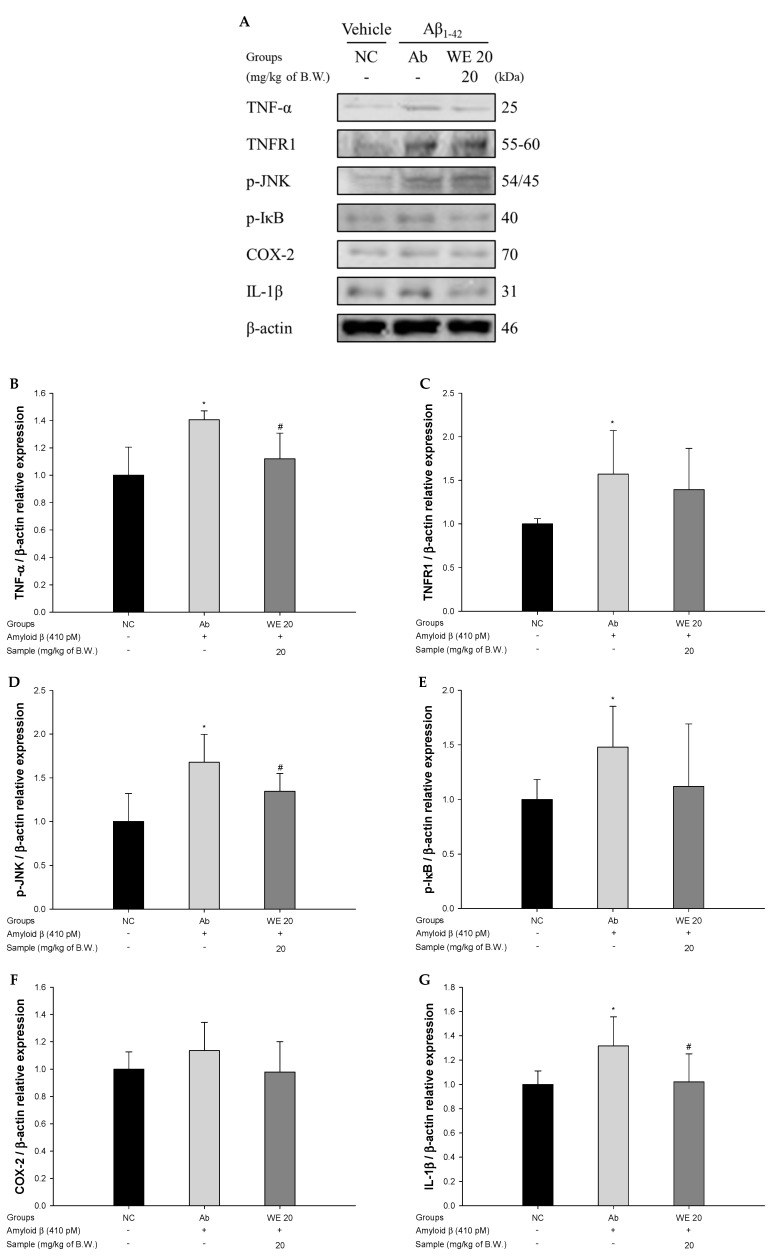
Protective effect of walnut (*Juglans regia* L.) extract on Aβ-induced neuro-inflammation: (**A**) protein expression levels; (**B**) representative Western blots for total protein and expression of tumor necrosis factor-alpha (TNF-α) (**B**), tumor necrosis factor receptor 1 (TNFR1) (**C**), phosphorylated c-Jun N-terminal kinase (p-JNK) (**D**), phosphorylated nuclear factor of kappa light polypeptide gene enhancer in B-cells inhibitor (p-IκB) (**E**), cyclooxygenase-2 (COX-2) (**F**), and interleukin 1 beta (IL-1β) (**G**) in mice brain tissues. Results shown are means ± SD (*n* = 3). Data are statistically represented at * = significantly different from the NC group; ^#^ = significantly different from Ab group, respectively; * and ^#^ *p* < 0.05.

To assess the protective effect of the walnut extract on Aβ_1-42_-induced mice via the regulation of neuroinflammation, the protein expressions of TNF-α, tumor necrosis factor receptor 1 (TNFR1), p-JNK, phosphorylated nuclear factor of kappa light polypeptide gene enhancer in B-cells inhibitor (p-IκB), cyclooxygenase-2 (COX-2), and IL-1β were measured (Figure 8). TNF-α, TNFR1, p-JNK, p-IκB, COX-2, and IL-1β in the Ab group were significantly overexpressed (40.64%, 57.13%, 67.81%, 47.86%, 13.54%, and 31.57%, respectively) compared to the NC group. However, the administration of the walnut extract decreased TNF-α, TNFR1, p-JNK, p-IκB, COX-2, and IL-1β levels (20.35%, 11.33%, 19.86%, 24.33%, 13.71%, and 22.39%, respectively) compared to the Ab group. In particular, expression levels of TNF-α, p-JNK, and IL-1β was significantly down-regulated compared to the Ab group.

### 3.8. Aβ-Related Akt Pathway

**Figure 9:**
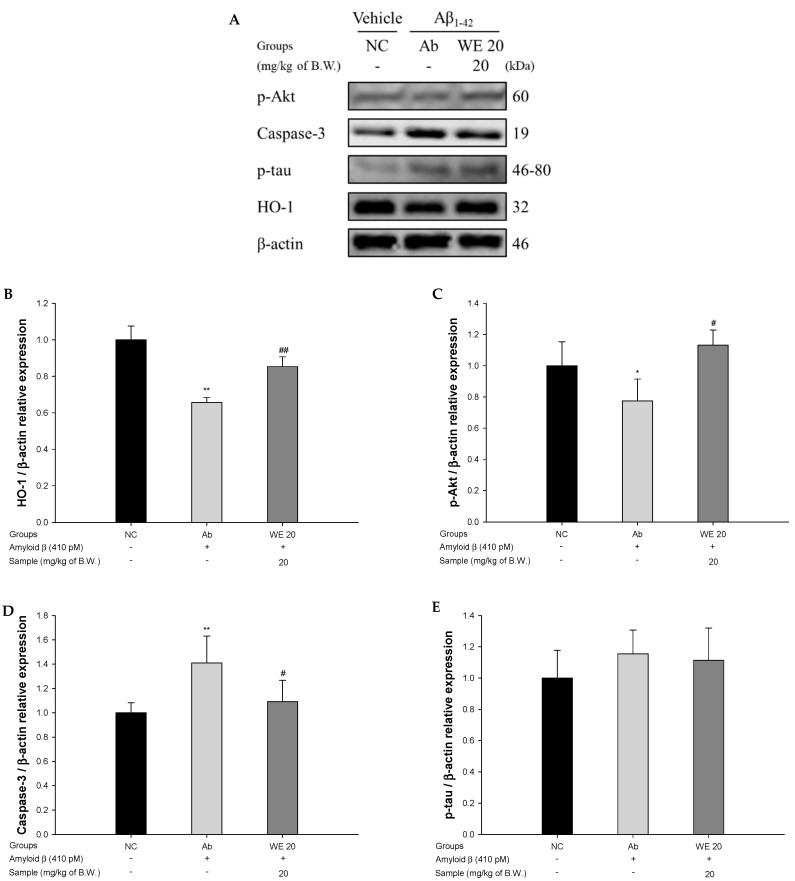
Protective effect of walnut (*Juglans regia* L.) extract on Aβ-induced, Aβ-related, protein kinase B (Akt) protein expression change: (**A**) protein expression levels; (**B**) representative Western blots for total protein and expression of phosphorylated Akt (p-Akt) (**B**), caspase-3 (**C**), hyperphosphorylated tau (p-tau), (**D**) and heme oxygenase-1 (HO-1) (**E**) in mice brain tissues. Results shown are means ± SD (*n* = 3). Data are statistically represented at * = significantly different from the NC group; ^#^ = significantly different from Ab group, respectively; * and ^#^ *p* < 0.05, ** and ^##^ *p* < 0.01.

To assess the protective effect of the walnut extract on Aβ_1-42_-induced mice via the improvement of Akt-related protein expression, the protein expressions of heme oxygenase-1 (HO-1), capase-3, and p-tau were measured (Figure 9). The expression levels of p-Akt and HO-1 in the Ab group were reduced (22.60% and 34.37%, respectively) compared to the NC group. On the other hand, the administration of the walnut extract restored the expression of p-Akt and HO-1 (46.19% and 30.00%, respectively) compared to the Ab group. Also, the level of capase-3 in the Ab group was significantly overexpressed (41.05%) compared to the NC group. However, the administration of the walnut extract significantly decreased the expression level of capase-3 (22.52%) compared to the Ab group. Also, the expression levels of p-tau for all the groups were not statistically different.

## 4. Discussion

AD is a degenerative disease caused by inflammatory responses and oxidative stress in the neuronal system. The causes of AD are known as oxidative stress and inflammatory cytokines induced by the aggregation of Aβ [30]. Oxidative stress causes the peroxidation of polyunsaturated fatty acids and the oxidation of proteins and DNA, and reduces antioxidants such as catalase, SOD, and GSH [31]. In particular, Aβ produces H_2_O_2_ and free radicals in hippocampal neurons, triggering the formation of aged glycosylation products, MDA, and neurofibrillary tangles [32]. In addition, Aβ indicates a decrease in BBB function and continuously stimulates the inflammatory response, leading to neuronal damage [33]. Therefore, this study was conducted to evaluate the anti-amnesic effect of walnut via the regulation of BBB function and anti-inflammatory effects in Aβ_1-42_-induced ICR mice.

Aβ leads to behavioral disorders and memory loss by damage to the hippocampus, amygdala, thalamus, and cerebellum in AD patients [34]. These areas are organically related to the processing of memory, consolidation of information between short-term and long-term memory, and decision making [35]. Aβ causes abnormalities in various brain functions through the generation of oxidative stress and inflammatory reactions, and eventually leads to cognitive impairment [34]. Therefore, it is important to evaluate cognitive dysfunction in the early stages of AD by conducting behavioral evaluation [14]. In various studies, i.c.v. Aβ injection has been reported to impair the memory and learning process, determined by evaluating a variety of experimental studies, such as novel object recognition and passive avoidance, as well as Y-maze and MWM tests [36,37,38,39]. Based on previous studies, cognitive and memory function was estimated in Aβ_1-42_-induced mice. The administration of walnut showed improvement of spatial learning and memory function compared to Aβ-injected mice. Also, walnut significantly improved short-term memory capacity and spatial learning ability. According to Wang et al., walnut intake improved lipopolysaccharide (LPS)-induced cognitive dysfunction through the regulation of pro-inflammatory mediators, such as NO and prostaglandin E2, and pro-inflammatory cytokines, such as TNF-α, IL-1β, and IL-6 [40]. Administration of walnut polypeptide maintains the shape of hippocampal CA3 pyramidal neurons through improvement of the cholinergic system and an increase in monoamines neurotransmitters, such as dopamine, norepinephrine, and serotonin [41]. This cognitive function improvement effect seems to be due to various ellagitannin contained in walnuts. Ellagitannin is decomposed and absorbed as ellagic acid or gallic acid through intestinal metabolism [42]. Similar to walnut, decomposed ellagitannin metabolites also ameliorate learning and memory deficits via the protective effect of the CA1 region of the hippocampus and the regulation of nuclear factor-kappa B (NF-κB), toll-like receptor 4 (TLR4), and nuclear factor (erythroid-derived 2)-like 2 (Nrf2) [43]. Based on these results, it is suggested that walnut extract containing various ellagitannins has a protective effect on cognitive dysfunctions in amyloidogenic mice.

Oxidative stress plays an important role in the pathogenesis of neuronal degeneration and death. This stress causes an imbalance between the production of ROS and the defense system, and ultimately leads to cerebral neuronal death, neurotransmitter loss, and AD [44]. In particular, brain tissue contains a large amount of unsaturated fatty acids and has a lower number of antioxidants, such as catalase and GSH, compared to other organs [45]. Therefore, since brain tissue has a structure that is vulnerable to external stress, it is necessary to consume functional food materials or antioxidants with a high protective effect against oxidative stress [46]. This study investigated the improvement of the antioxidant system by the administration of walnut extract in Aβ_1-42_-induced mice brains. Previous studies have reported that the intake of walnuts significantly lowered the content of MDA, inhibited the reduction of SOD, and reduced GSH. Similar to this study, walnut consumption restored SOD, glutathione peroxidase (GPx), and catalase activities in scopolamine-induced cognitive impairment in rats [47]. In addition, walnut contains large amounts of polyphenols, such as digalloylglucose, ellagic acid, glansreginin A, rutin, pedunculagin, casuarictin, casuarinin, and glansreginin B. Walnut with these compounds, with high SOD-like activity and DPPH scavenging activity, ameliorated liver and serum MDA levels, as well as SOD and CAT activity in diabetic rats [48,49,50]. Based on these results, it is considered that the intake of walnuts suppressed the progress of antioxidant system damage, due to the various polyphenols with excellent antioxidant activity, and can be a material that helps improve cognitive function. 

Abnormality of the cholinergic system in AD patients is closely related to the amyloid mechanism [51]. In general, ACh regulated by AChE and butyrylcholinesterase (BChE) in brain tissue plays an important role in neurotransmission. However, in AD patients, decreased ACh levels and the increased activity of AChE and BChE were observed [52]. Additionally, amyloid peptides inhibit the movement of choline in neuronal synapses and reduces the neurotransmission function by inhibiting the release of ACh in cells [53]. In addition, amyloid peptides have a high affinity for ACh receptors and AChE by forming a complex with them. Since the Aβ–AChE complex has a more stable structure than normal AChE, this complex excessively decomposes ACh and inhibits the activity of ACh receptors. Also, this complex increases the rate of Aβ fibril formation and neurotoxicity. Since this complex has a very stable structure, it shows strong neurotoxicity and promotes synaptic damage [54]. Based on these studies, walnut extract restored the contents of ACh, suppressed the activity of AChE in the brain tissue, and increased the expression level of ChAT. It has been reported that walnuts exhibit excellent AChE inhibitory activity when compared with various natural food ingredients, such as onion (*Alium cepa* L.), garlic (*Allium sativum*), kale (*Brassica oleracea* var. *acephala*), and broccoli (*Brassica oleracea* var. *italica*) [55]. In addition, walnuts contain large amounts of choline, which is important for acetylcholine synthesis and cholinergic neurotransmission as a source of sphingomyelin and phosphatidylcholine [56]. Walnut is reported to be a cholesterol-lowering material containing various kinds of phytosterols, such as beta-sitosterol, campesterol, and stigmasterol [57]. These phytosterols modulate the molecular processes of Aβ produced by an amyloid precursor protein (APP) [58]. Therefore, the consumption of walnuts will regulate the imbalanced metabolism of acetylcholine and effectively protect cognitive and memory functions.

Aβ easily binds to the mitochondrial membrane, and the complexed mitochondrial Aβ causes lipid peroxidation and ROS, resulting in an abnormal state of mitochondria [8]. In particular, mitochondria degraded by Aβ significantly reduces ATP production and suppresses the energy supply to neuronal cells [59]. In addition, Aβ peptides are aggregated as a form of oligomer, with a sharp shape at the end of the synapse. Aggregated Aβ oligomers can damage organelles like the mitochondria [60]. Damaged mitochondria cause a continuous deficit to the electron transport system, resulting in the dysfunction of calcium ion homeostasis and destruction of MMP. These mitochondrial disorders limit the energy supply to neurons and eventually promote neuronal death [61]. Therefore, the protective effect of walnut against mitochondrial dysfunction in Aβ_1-42_-induced mice was confirmed, and walnut extract showed improved mitochondrial function. According to a previous study, walnuts lowered the ROS content, disruption of MMP, and mitochondrial swelling when exposed to Aβ peptides. It inhibits the release of cytochrome C, which induces apoptosis in mitochondria [60]. In addition, walnut (*Juglans mandshurica* Maxim.) inhibits the production of mitochondrial ROS and reduces ATP content through the regulation of Akt/mammalian targets of serine/threonine protein kinase rapamycin (mTOR) signals and LC3-II/LC3-I levels in Aβ_25–35_-induced PC12 cells [61]. Therefore, similar to these results, walnut has a protective effect on mitochondrial abnormalities through ROS scavenging activity, as well as the protection of mitochondrial effects on cognitive function by improving neuronal energy metabolism.

An imbalance between the production and elimination of Aβ in cerebral small arteries leads to amyloid angiopathy aggregating the Aβ peptides in brain tissue [62]. Accumulation of Aβ leads to impaired BBB function related to Aβ clearance. This dysfunction results in a reduction in capillary diameter, and ultimately contributes to excessive neuroinflammation and the onset of apoptosis [63]. Originally, BBB is maintained by tight junction complexes consisting of occludin, claudin-1/3/5, ZO-1/2/3, and actin cytoskeletons that link transmembrane proteins, and these proteins are linked to accessory cytoplasmic proteins of members of the zona occludens family, including ZO-1/2/3 [64]. The increased production of Aβ promotes the degradation of tight junctions and increases BBB permeability through downregulation of ZO-1, claudin-5, and occludin [62]. The decrease in the expression of these proteins does not normally perform the tight junction function, and increases Aβ toxicity and neuroinflammation [63]. Eventually, Aβ not only generates toxicity by itself, but also does not eliminate the inflammatory response generated by external Aβ due to deficit of the BBB, resulting in continuous neuronal cell damage [62]. Therefore, the expression of ZO-1 and occludin was measured to confirm the protective effect of walnut extract against BBB damage caused by Aβ injection. Walnut extract increased the expression levels of ZO-1 and occludin in Aβ_1-42_-induced mice brain tissue. According to Farbood et al., ellagic acid as the decomposed form of ellagitannin improved the maintenance of the BBB and lowered the content of IL-1β and IL-6 in a traumatic hippocampal injury rat model [65]. In addition, gallic acid, a metabolite of ellagitannin, improved BBB dysfunction against exposure to dusty particulate matter, improving oxidative stress damage in the brain [66]. Therefore, it is estimated that damage to the BBB could be improved by walnut and its metabolites, such as ellagic acid and gallic acid.

Along with the breakdown of the BBB, Aβ produces oxidative stress and increases the neuroinflammatory response in the brain tissue [6]. Aβ promotes an increase in TNF-α levels and the activity of p-JNK, inducing phosphorylation of serine residues of IRS-1 (IRS-1pSer) instead of tyrosine residues [67]. IRS-1pSer lowers the activity of insulin degrading enzyme (IDE) and neprilysin (NEP) as the Aβ accumulation inhibitory enzymes, and continuously induces Aβ aggregation and toxicity [9]. During this process, a large number of inflammatory cytokines, such as IL-1β, IL-6, and TNF-α are expressed in neuronal cells. This increased inflammatory response affects the survival of neurons, and eventually initiates AD [68]. Therefore, the protective effect of walnut extract against inflammatory damage caused by an injection of Aβ was confirmed by measuring the expression of neuro-inflammatory protein. The intake of walnuts exhibited autophagy regulation through the inhibition of mTOR phosphorylation, the upregulation of autophagy-related 7 (ATG7) and Beclin 1, and the conversion of microtubule-associated protein 1B light chain 3 (MAP1BLC3) in aged rats [69]. Walnut polyunsaturated fatty acids showed improvement of inflammatory response in LPS-induced hippocampal death and calcium dysregulation [70]. Also, glansreginin A, one of the bioactive substances in walnuts, has been shown to inhibit abnormal behavior and the overactivation of microglia in LPS-induced mice [71]. According to previous studies, walnut showed significant improvement of inflammatory responses by various stresses. Similar to these studies, walnut has an anti-therapeutic effect on Aβ_1-42_-induced cerebral neuroinflammation via regulation of the inflammatory pathway. Also, the inflammatory response caused by Aβ is regulated by the BBB, but the damage to the BBB increases the inflammatory response in neuronal cells. On the other hand, walnut extract can significantly suppress the inflammatory response that appears from the self-toxicity of Aβ and damage to the BBB.

p-Akt expression related to cell survival easily inhibits the excessive inflammatory response. The down-expressed p-Akt affects the abnormalities of neurons through various pathways, such as the apoptosis pathway, production of neurofibrillary tangles (NFTs), and damaged antioxidant defense systems [72]. Excessive expression of p-JNK indicates a decrease in p-Akt, and continuously promotes the activity of p53 [7]. Activated p53 increases the ratio of Bcl-2-associated X protein (BAX)/Bcl-2 and the release of mitochondrial cytochrome C. The released cytochrome C stimulates an increase in the expression of caspase-3 and caspase-9, and this caspase cascade induces apoptosis [73]. Therefore, the regulation of p-Akt is ultimately associated with the inhibition of apoptosis, and this study suggests that walnut extract has a neuroprotective effect by inhibiting the apoptosis of neurons through the regulation of p-Akt and caspase-3. In addition, a decrease in p-Akt is associated with the expression of tau protein, regulating axonal transport, and the stabilization of microtubules in neurons. Normal Akt activity regulates the activation of glycogen synthase kinase-3β (GSK-3β), inhibiting the phosphorylation of tau related to stabilized microtubules [74]. However, reduced Akt activity induces tau phosphorylation without inhibiting GSK-3β activation [75]. This p-tau is continuously combined, with the produced tau as a dimer and oligomers forming on the axis of neurons, and ultimately leads to a decrease in synaptic plasticity and neuronal cell death by producing NFTs [74]. Therefore, walnut extract can inhibit neuronal death by inhibiting fibril formation of p-tau through the amelioration of p-Akt. The decrease in p-Akt also affects the reduction of the antioxidant system’s ability to eliminate ROS and oxidative stress [72]. p-Akt increases the expression of Nrf2 from Kelch-like, ECH-associated protein 1 (keap1), and the generated Nrf2 increases the expression of HO-1, NAD(P)H quinone oxidoreductase (NQO1), and SOD protecting the antioxidant system [76]. However, a decrease in p-Akt inhibits the normal function of the neuronal survival pathway, and accumulated oxidative stress leads to neuronal cell death [72]. This inhibition of a decrease in p-Akt is related to the protective effect of neurons. Therefore, this study confirmed the expression of caspase-3, p-tau, and HO-1 as the various sub-factors of Akt, and walnut intake showed an ameliorating effect on these proteins. According to Liu et al., walnut peptide inhibited the expression of cytochrome C, caspase-9, caspase-3, and poly (ADP-ribose) polymerase (PARP) related to neuronal cell death, and upregulated the expression of phosphorylated cAMP response element-binding protein (p-CREB) and synaptophysin to protect neuronal cells [77]. In addition, walnut oligopeptides reduced Aβ levels and p-tau production, and improved the expression of PI3K and the ratio of p-Akt/Akt in the aged SAMP8 mice hippocampus [78]. In addition, walnut (*Juglans mandshurica* Maxim.) leaf extract increased the levels of HO-1 and Nrf2 by increasing matrix metalloproteinase-1 (MMP-1) and inhibiting the extracellular signal-regulated kinase (ERK), p38 and JNK in H_2_O_2_-induced skin fibroblasts [79]. Ellagic acid, as one of the walnut metabolites, increases the expression of Nrf2 and HO-1 in the aorta, and it can protect cerebral neurons through the improvement of the antioxidant system of the BBB [80]. Therefore, it is considered that walnut extract inhibits neuronal cell death through the Akt pathway in amyloidogenic mice. However, the identification of additional peptides contained in walnuts will have to be performed. In some experiments, the effect in the WE 10 group was not significant. However, administration of walnut extract significantly improved cognitive function and the neuro-protective effect. In conclusion, it can be confirmed that walnut extract has a summative effect on Aβ-induced pathology and cognitive function improvement by improving the p-Akt pathway and inflammatory response via the ameliorating effect of BBB function.

## 5. Conclusions

**Figure 10:**
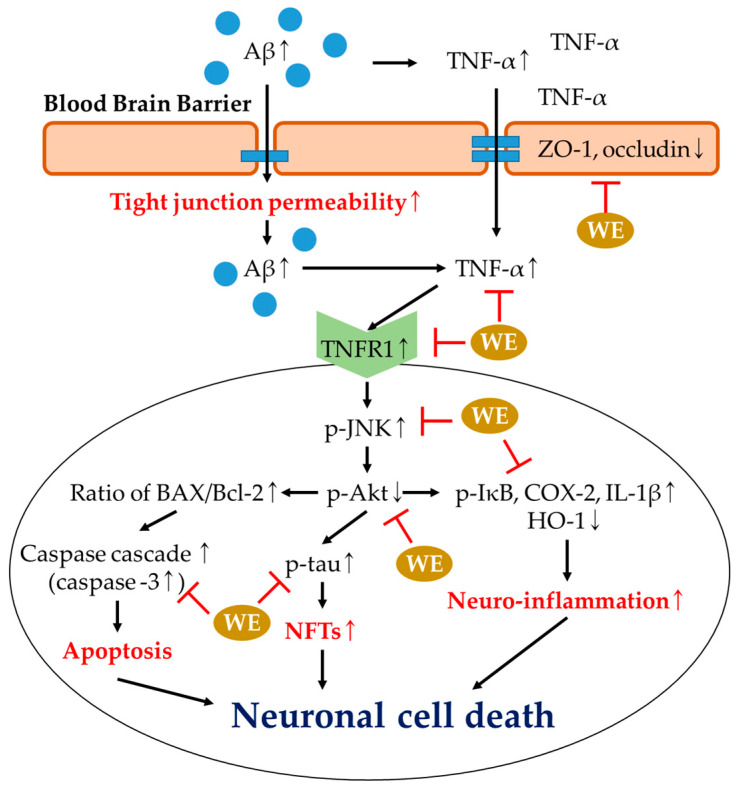
A schematic illustration shows the ameliorating effect of walnut extract (WE) in Aβ_1-42_-induced institute of cancer research (ICR) mice via regulation of the blood–brain barrier (BBB) function and neuro-inflammatory response.

In summary, these results suggest that walnut extract, including its various bioactive compounds, has a significant protective effect on memory loss and cognitive dysfunction in Aβ_1-42_-injected ICR mice. Walnut extract protected the cholinergic and antioxidant system, and restored mitochondrial dysfunction. Also, walnut extract restored Aβ_1-42_-induced BBB dysfunction and had an anti-inflammatory effect via the Aβ-induced Akt pathway. Through this study, it was confirmed that the intake of natural products can reduce oxidative stress in brain tissue and improve cognitive function through improvement of BBB function and inflammatory response. In conclusion, it is suggested that walnut extract could be used as a material for functional food to ameliorate memory loss and cognitive dysfunction via the regulation of BBB functions and neuroinflammation (Figure 10).

## Figures and Tables

**Table 1:** List of antibodies and their information used in this study.

Antibody	Catalog	Conc.	Manufacturer
β-actin	sc-69879	1:1000	Santa Cruz Biotech (Dallas, TX, United States)
AChE	sc-373901	1:1000	Santa Cruz Biotech (Dallas, TX, United States)
p-JNK	sc-6254	1:1000	Santa Cruz Biotech (Dallas, TX, United States)
p-Akt	sc-514032	1:1000	Santa Cruz Biotech (Dallas, TX, United States)
p-tau	sc-12952	1:1000	Santa Cruz Biotech (Dallas, TX, United States)
ZO-1	sc-33725	1:1000	Santa Cruz Biotech (Dallas, TX, United States)
Occludin	sc-133256	1:1000	Santa Cruz Biotech (Dallas, TX, United States)
HO-1	sc-136960	1:1000	Santa Cruz Biotech (Dallas, TX, United States)
COX-2	sc-376861	1:1000	Santa Cruz Biotech (Dallas, TX, United States)
p-IκB	sc-8404	1:1000	Santa Cruz Biotech (Dallas, TX, United States)
IL-1β	sc-4592	1:1000	Santa Cruz Biotech (Dallas, TX, United States)
ChAT	20747-1AP	1:1000	Bioneer (Daejeon, Korea)
TNF-α	5178SC	1:1000	Cell Signaling Tech (Danvers, MA, United States)
Caspase-3	CSB-PA05689A0Rb	1:1000	Cusabio (Hubei, China)
TNFR1	CSB-PA621879EA01HU	1:1000	Cusabio (Hubei, China)

AChE, acetylcholinesterase; p-JNK, phosphorylated c-Jun N-terminal kinase; p-Akt, phosphorylated protein kinase B; ZO-1, zonula occludens-1; HO-1, heme oxygenase-1; COX-2, cyclooxygenase-2; p-IκB, phosphorylated nuclear factor of kappa light polypeptide gene enhancer in B-cells inhibitor; IL-1β, interleukin 1 beta; ChAT, choline acetyltransferase; TNF-α, tumor necrosis factor-alpha; TNFR1, tumor necrosis factor receptor 1.

**Table 2:** Compounds identified from walnut (*Juglans regia* L.) extract.

No.	RT ^a^	Parent Ion ^b^	MS^2^ Ions ^c^	Compound
	(min)	(*m*/*z*)	(*m*/*z*)	
1	2.55	783	481, **301**, 275	Pedunculagin/casuariin isomer (bis-HHDP–glucose)
2	2.71	783	481, **301**, 275	Pedunculagin/casuariin isomer (bis-HHDP–glucose)
3	2.88	951	907, 783, **301**, 275	Praecoxin A/platycariin isomer (trigalloyl-HHDP–glucose)
4	2.90	785	633, 483, **301**, 275	Tellimagrandin I isomer (digalloyl-HHDP–glucose)
5	3.03	935	785, 633, 481, **301**, 275	Casuarinin/casuarictin isomer
6	3.06	433	**301**	Ellagic acid pentoside
7	3.22	1085	633, 451, **301**	Eucalbanin A/cornusiin B isomer
8	3.39	592	**567**, 403, 343, 283, 241, 197	Glansreginin A

^a^ RT means retention time. ^b^ Ions are presented at *m*/*z* [M - H]^−^. ^c^ Bold indicates the main fragment of MS^2^.

## Supplementary Materials

The following are available online at https://www.mdpi.com/2076-3921/9/10/976/s1, Figure S1: 2,2′-azino-bis(3-ethylbenzothiazoline-6-sulfonic acid) (ABTS) (A), 2,2-diphenyl-1-picrylhydrazyl (DPPH) (B) radical scavenging activity, and ferric reducing antioxidant power (FRAP) (C) of ethanolic extracts from Korean domestic cultivars (Gimcheon 1ho, Geumgok, and Geumreung) and imported from oversea cultivars (China and United States), Figure S2: Cell viability of walnuts (*Juglans regia* L.; Geumgok cultivar) on H_2_O_2_-induced cytotoxicity in PC12 cells (A) and MC-IXC cells (B).
