# Supplementary material for: Anti-Amnesic Effect of Walnut via the Regulation of BBB Function and Neuro-Inflammation in Aβ1-42-Induced Mice
Source: Antioxidants (Basel). 2020 Oct 12;9(10):976. doi: 10.3390/antiox9100976 (PMC7600148; doi:10.3390/antiox9100976)
Supplement: Supplementary file 1 [file antioxidants-09-00976-s001.pdf]

Supplementary materials

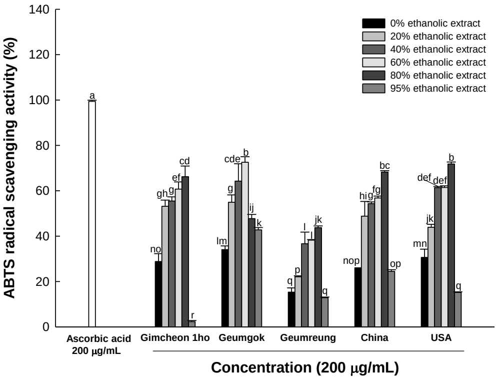

(**A**)

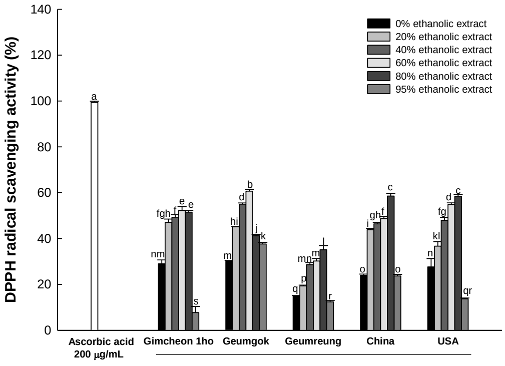

(**B**)

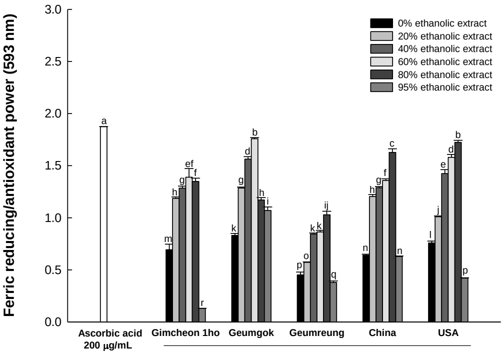

(**C**)

Figure 1. ABTS (A), DPPH (B) radical scavenging activity and FRAP (C) of ethanolic extracts from korean domestic cultivars (Gimcheon 1ho, Geumgok and Geumreung) and imported from oversea cultivars (China and USA). Results shown are mean ± SD ($n = 3$). Data were statistically considered at $p < 0.05$, and different small letters represent statistical differences.

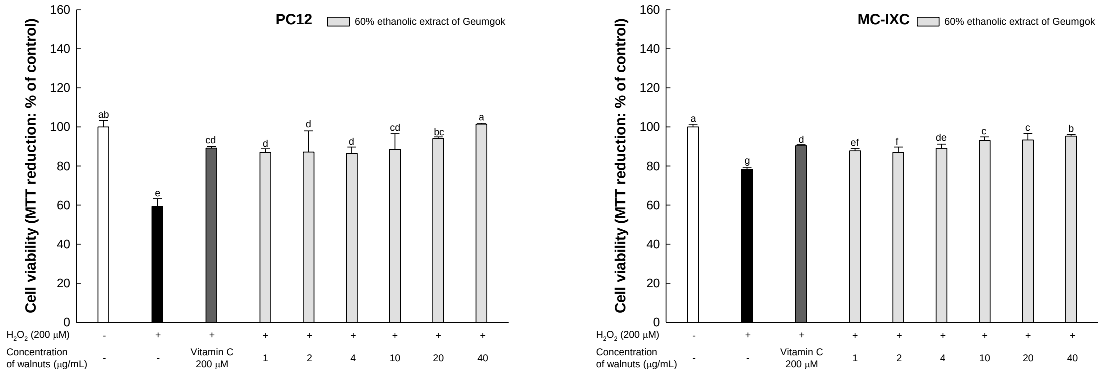

(**A**) (**B**)

Figure 2. Cell viability of walnuts (*Juglans regia* L.; Geumgok) on H_2O_2-induced cytotoxicity in PC12 cells (A) and MC-IXC cells (B). Results shown are mean ± SD ($n = 3$). Data were statistically considered at $p < 0.05$, and different small letters represent statistical differences.
